# Draft Genome Sequence of *Psychrobacter piscatorii* Strain LQ58, a Psychrotolerant Bacterium Isolated from a Deep-Sea Hydrothermal Vent

**DOI:** 10.1128/genomeA.00044-16

**Published:** 2016-03-03

**Authors:** Meixian Zhou, Binbin Dong, Qing Liu

**Affiliations:** a Key Laboratory of Marine Genetic Resources, Third Institute of Oceanography, State Oceanic Administration, Xiamen, China; b Key Laboratory of Marine Genetic Resources of Fujian Province, Xiamen, China

## Abstract

Here, we report the 3.1-Mb draft genome sequence of *Psychrobacter piscatorii* strain LQ58, isolated from a deep-sea hydrothermal vent on the East Pacific Rise. The sequence will provide further insight into the environmental adaptation of psychrotolerant bacteria and the development of novel cold-active enzymes for industrial application.

## GENOME ANNOUNCEMENT

Members of the genus *Psychrobacter* are psychrotolerant or psychrophilic bacteria, which are widely distributed in marine and terrestrial environments (1–3). They have been considered good sources of cold-active enzymes for industrial and environmental uses (4–6). *Psychrobacter piscatorii* strain LQ58 (=MCCC 1A10701) is an aerobic, psychrotolerant, chemoorganotrophic bacterium, isolated from a deep-sea hydrothermal vent sediment sample collected on the East Pacific Rise at a depth of 2,906 m (3.10 S, 102.55 W). Growth was observed at temperatures between 0°C and 35°C with an optimum at 22°C in the presence of 2% NaCl. Strain LQ58 showed highest 16S rDNA sequence similarity with *P. piscatorii* T-3-2 (99.3%) (7), followed by *Psychrobacter aquaticus* CMS56 (98.6%) (8). *P. piscatorii* was reported to exhibit an extraordinarily high catalase activity (9). To date, there is no reference *P. piscatorii* genome sequence available publicly. Here, we report the draft genome sequence of strain LQ58, the first released *P. piscatorii* genome sequence, which will provide further insight into the environmental adaptation of psychrotolerant bacteria and the development of novel cold-active enzymes for industrial application.

Extraction of genomic DNA from strain LQ58 was carried out using a bacterial DNA kit (Omega) according to the manufacturer’s instructions. The genome of strain LQ58 was sequenced by whole-genome shotgun sequencing using the Illumina MiSeq system at Shanghai Majorbio Bio-pharm Technology Co., Ltd. (Shanghai, China). A total of 804,651,728 reads were generated, representing an approximately 260-fold coverage of the genome. These reads were assembled using SOAPdenovo version 2.0 (10) and resulted in 139 contigs with an *N*_50_ of 47,940 bp. The size of the longest contig was 149,861 bp. The draft genome of strain LQ58 was analyzed and annotated using the NCBI Prokaryotic Genome Annotation Pipeline (PGAP) (http://www.ncbi.nlm.nih.gov/genome/annotation_prok). Genes of interest were manually evaluated.

The draft genome sequence of strain LQ58 was 3,089,314 bp in length, with a G+C content of 44.1%. The genome contains 2,676 predicted open reading frames and 2,562 predicted protein-coding sequences). There were 33 tRNA genes, 3 rRNA genes, and 1 noncoding RNA (ncRNA) gene predicted from this assembly.

*In silico* DNA-DNA hybridization (DDH) and average nucleotide identity values of LQ58 against *P. aquaticus* CMS56 were 22.6 ± 2.36% and 79.3% (11), respectively, which was significantly lower than the recommended cutoff value for species delineation (12, 13).

Sequence analysis revealed that the LQ58 genome encodes 37 lipolytic enzymes, 58 peptidases, 2 formate dehydrogenases, 12 alcohol dehydrogenases, and 7 glycosyltransferases for potential biotechnology applications. LQ58 genome also encodes 3 cold-shock proteins (CspA, CspC and CspD), 1 capsular polysaccharide synthesis gene cluster, and 1 GroES-GroEL operon associated with cold stress (14). In addition, compared with other sequenced *Psychrobacter* genomes, LQ58 contains more catalase genes, including 1 HPII-like monofunctional catalase gene and 1 HPI-like bifunctional catalase gene (15), which may be favorable for its adaptation to an oxidative environment.

### Nucleotide sequence accession numbers.

This whole-genome shotgun project has been deposited at DDBJ/EMBL/GenBank under the accession number LNDJ00000000. The version described in this paper is the first version, LNDJ01000000.
